# The nuclear import of ribosomal proteins is regulated by mTOR

**DOI:** 10.18632/oncotarget.2473

**Published:** 2014-10-03

**Authors:** Dubek Kazyken, Yelimbek Kaz, Vladimir Kiyan, Assylbek A. Zhylkibayev, Chien-Hung Chen, Nitin K. Agarwal, Dos D. Sarbassov

**Affiliations:** ^1^ Department of Molecular and Cellular Oncology, University of Texas M. D. Anderson Cancer Center, Houston, TX 77030, USA; ^2^ Department of Natural Sciences, The L.N. Gumilyov Eurasian National University, Astana, 010008, Kazakhstan; ^3^ The University of Texas Graduate School of Biomedical Sciences at Houston, TX 77030, USA

**Keywords:** mTOR (mechanistic target of rapamycin), kinase, ribosomal proteins, nuclear import

## Abstract

Mechanistic target of rapamycin (mTOR) is a central component of the essential signaling pathway that regulates cell growth and proliferation by controlling anabolic processes in cells. mTOR exists in two distinct mTOR complexes known as mTORC1 and mTORC2 that reside mostly in cytoplasm. In our study, the biochemical characterization of mTOR led to discovery of its novel localization on nuclear envelope where it associates with a critical regulator of nuclear import Ran Binding Protein 2 (RanBP2). We show that association of mTOR with RanBP2 is dependent on the mTOR kinase activity that regulates the nuclear import of ribosomal proteins. The mTOR kinase inhibitors within thirty minutes caused a substantial decrease of ribosomal proteins in the nuclear but not cytoplasmic fraction. Detection of a nuclear accumulation of the GFP-tagged ribosomal protein rpL7a also indicated its dependence on the mTOR kinase activity. The nuclear abundance of ribosomal proteins was not affected by inhibition of mTOR Complex 1 (mTORC1) by rapamycin or deficiency of mTORC2, suggesting a distinctive role of the nuclear envelope mTOR complex in the nuclear import. Thus, we identified that mTOR in association with RanBP2 mediates the active nuclear import of ribosomal proteins.

## INTRODUCTION

Accumulation of cellular mass is a fundamental biological process known as cell growth. The highly conserved and essential mechanistic target of rapamycin (mTOR) pathway has been defined as a crucial regulator of cell growth and proliferation by controlling anabolic processes in cells. The mTOR pathway regulates these processes according to diverse environmental cues by integrating the regulatory inputs such as nutrients, growth factors, energy, and stress [1–3].

mTOR, as a central component of this pathway, functions as the protein kinase by forming two distinct multi-protein complexes. The mTOR Complex 1 (mTORC1), defined by the mTOR interacting protein raptor, is a nutrient-sensitive complex that regulates cell growth and cell size. mTORC1 controls major anabolic processes by promoting protein synthesis, lipogenesis, and energy metabolism and also by inhibiting autophagy. The second complex of mTOR, known as mTORC2, is assembled by association of mTOR with rictor and SIN1 that functions as the regulatory kinase of Akt, PKCα, and SGK1 and regulates cell proliferation [3]. Evolutionary, mTORC1 is the most conserved component of mTOR signaling and exists in all eukaryotes, while mTORC2 has been identified in yeast and animals but its essential components rictor and SIN1 have not been identified in plants [4, 5].

Regulation of protein synthesis is a key functional role of mTOR signaling [6]. mTOR controls protein synthesis by regulation of the initiation and elongation factors of translation. Translational initiation is one of the rate limiting steps in protein synthesis that requires assembly of the eukaryotic translational initiation factor 4F (eIF4F) complex on the 5′ cap structure of mRNA. The eIF4F complex is assembled by three components, known as eIF4E, eIF4G, and eIF4A. The inhibitory 4E binding protein 1 (4E-BP1) prevents assembly of this complex by binding to one of its components eIF4E. mTORC1 restrains the inhibitory function of 4E-BP1 by phosphorylation and promotes assembly of the translational initiation complex eIF4F. In addition, mTORC1 regulates translation by phosphorylation and activation of S6 Kinases 1 and 2 (S6K1 and 2). Activated S6K1 phosphorylates or binds several proteins including eukaryotic elongation factor 2 kinase (eEF2K), S6K1 Aly/REF-like target (SKAR), 80 kDa nuclear cap-binding protein (CBP80) and eIF4B and promotes translation by regulating its initiation and elongation [3, 7].

The recent studies also relate mTORC2 to protein synthesis, but in this case, as its downstream effector. It has been reported that the growth factor-dependent activation of mTORC2 takes place in association with ribosomes [8] and it phosphorylates its substrate Akt on Thr-450 site as a co-translational event [9] indicating that the functional activity of mTORC2 is coupled to protein synthesis. Moreover, mTORC2 is localized predominantly in endoplasmic reticulum (ER) [10], the organelle associated with ribosomes, and the kinase activity of mTORC2 is sensitive to ER stress [11]. Thus, both mTORC1 and mTORC2 represent mTOR signaling as the upstream and downstream regulators of protein synthesis.

Besides the regulation of translation, mTOR also controls protein synthesis by regulating the biogenesis of ribosomes. The studies in yeast and mammals have demonstrated that the nutrient-dependent ribosomal biogenesis is mediated by mTOR signaling [12]. mTOR regulates the synthesis of ribosomal components by promoting expression and processing of pre-ribosomal RNA, expression of ribosomal proteins, and synthesis of 5S ribosomal RNA. Ribosomal biogenesis is a complex process [13, 14] driven not only by the rate of synthesis of ribosomal components but also by the nuclear import of ribosomal proteins [15, 16], assembly of ribosomes in nucleolus [17], and their export to cytoplasm [18]. mTOR, as a key regulator of anabolic processes, controls ribosomal biogenesis [12], though this functional role of mTOR remains poorly characterized. Here, we present evidence that mTOR in association with the nuclear pore component regulates the nuclear import of ribosomal proteins.

## RESULTS

### Detection of a novel mTOR complex in the salt extractable nuclear fraction

**Figure 1:**
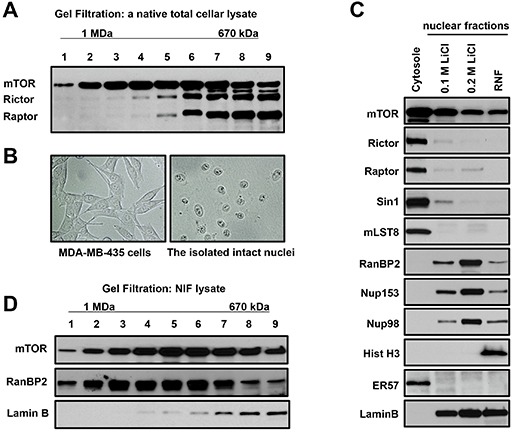
A novel mTOR complex is distinct from mTORC1 and mTORC2 by size and sub-cellular localization **(A)** mTOR exists in a complex larger than mTORC1 and mTORC2. The Sephacryl S-500 gel-filtration profile of the total cell lysate prepared from MDA-MB-435 cells has been analyzed by immunoblotting with the indicated antibodies. mTOR is eluted in the fractions corresponding to a complex larger than mTORC1 and mTORC2 (Fractions 1-4). Molecular size was calculated by creating retention/molecular size curve. **(B)** Intact nuclei are purified by the isotonic buffer. Phase contrast images of MDA-MB435 cells before sub-cellular fractionation (a left panel) and nuclei after fractionation (a right panel). **(C)** mTOR is detected in the cytoplasmic and nuclear fractions but raptor, rictor, Sin1, and mLST8 are detected predominantly only in the cytoplasmic fraction. The cytoplasmic and nuclear sub-fractionation of MDA-MA-435 cells has been performed in isotonic buffer to preserve nuclei. Nuclei were fractionated by a sequential extraction with the salt containing buffers and indicated as the Salt Extractible Nuclear Fraction (SENF) containing 0.1 M LiCl and 0.2 M LiCl. Following the salt extraction, the Residual Nuclear Fraction (RNF) has been obtained by lysis in 8 M Urea buffer. The extracts were analyzed as described in (A). **(D)** mTOR co-elutes with a Nuclear Pore Complex (NPC) protein RanBP2 in the nuclear extract. The nuclear extract obtained by 0.1 M LiCl was fractionated using the S-500 Sephacryl gel column and analyzed as described in (A).

In previous studies both mTOR complexes have been identified by affinity purification [19–21]. To examine a size of native mTOR complexes, we analyzed the cellular lysates obtained without detergent through several freeze/thaw cycles by exploiting the gel-filtration chromatography. We observed that mTORC1 [19] and mTORC2 [20, 21] were detected in the same fractions corresponding to molecular weight particles in a range of 670 kDa as detected by co-elution of mTOR with raptor (mTORC1) and with rictor (mTORC2) as shown in Figure 1A. Interestingly, we also isolated the fractions with larger size particles containing a considerable amount of mTOR without raptor and rictor. Based on this finding we hypothesized that, besides mTORC1 and mTORC2, mTOR also exists in a novel complex.

We further pursued isolation of a novel mTOR complex by performing the sub-cellular fractionation. To purify intact nuclei, we have optimized the cell lysis condition in isotonic buffer with a low concentration of detergent without applying mechanical force, since the commonly used nuclear purification carried in hypotonic conditions is associated with swelling of nuclei and distortion of the nuclear/cytoplasmic gradient [22, 23]. Following sub-cellular fractionation of cells in the isotonic buffer, we observed the intact nuclei as shown by the images of cells and nuclei after fractionation (Fig. 1B). We found that mTOR is detected in the cytoplasmic and nuclear fractions, but the mTOR interacting proteins rictor, raptor, and mLST8 were found mostly in the cytoplasmic fraction (Fig. 1C). It indicates that the cytoplasmic fraction as detected by a marker ERp57 [24] contains the essential components of mTORC1 and mTORC2 (raptor, rictor, and mLST8) [3]. The purified nuclei were further extracted sequentially by the buffers containing 0.1 M and 0.2 M LiCl. The obtained nuclear extracts indicate that a higher (0.2 M) salt concentration is more effective in extraction of mTOR (Fig. 1C). Based on this observation, we carried out our study by extracting isolated nuclei with the buffer containing 0.2 M LiCl and we referred to this nuclear fraction as the Salt Extractable Nuclear Fraction (SENF). Besides mTOR, the 0.2 M salt extraction was also effective in stripping the Nuclear Pore Complex (NPC) proteins [25] nucleoporin 153 (Nup153), Ran Binding Protein 2 (RanBP2, also known as Nup358), and Nup98, where a substantial amount of the nuclear envelope protein lamin B remained in the Residual Nuclear Fraction (RNF). After the salt extraction, nuclei retain their structure as detected by presence of histone H3 only in RNF. We find that the known mTOR interacting proteins rictor, raptor and mLST8 that form two distinct complexes defined as mTORC1 and mTORC2 are barely detectable in the nuclear fractions (Fig. 1C). The gel filtration analysis also indicated that mTOR extracted from nuclei remained in a large complex that is co-eluted with the nucleoporin RanBP2 but not lamin B (Fig. 1D) Thus, we show that a novel mTOR complex is distinct from mTORC1 and mTORC2 [3] by its size and sub-cellular localization.

### mTOR associates and is co-localized with the nuclear pore component RanBP2

**Figure 2:**
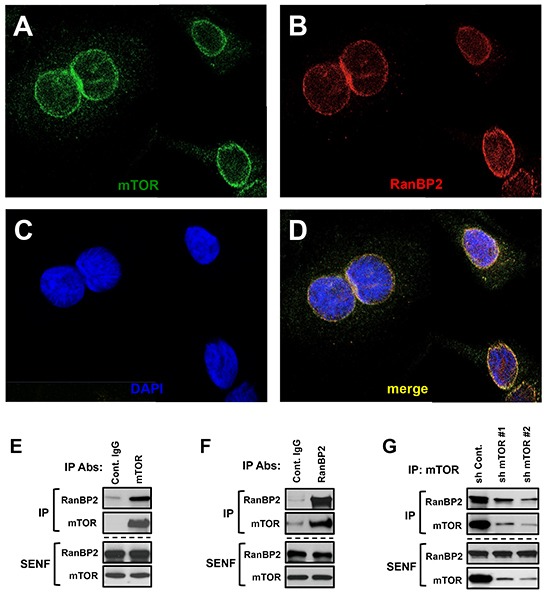
mTOR and RanBP2 proteins form a complex on the nuclear envelope (A-D) The co-localization of mTOR and RanBP2 on the nuclear envelope. Confocal images of mTOR and RanBP2 co-localization: **(A)** Immunostaining of mTOR in nuclei. **(B)** Immunostaining of RanBP2 in nuclei. **(C)** Nuclear staining by DAPI. **(D)** The merged images of mTOR and RanBP2. E-G, The immunoprecipitations of RanBP2 or mTOR from the salt extractable nuclear fraction (SENF) obtained from different human cancer cell lines have been analyzed by immunoblotting with the indicated antibodies. **(E)** mTOR is co-purified with RanBP2 and ***(F)*** RanBP2 is co-purified with mTOR from extracts of MDA-MB-435 cells. **(G)** Co-purification of RanBP2 with mTOR is specific: the shRNAs targeting luciferase (control) or mTOR were lentivirally transduced into MDA-MB-435 cells. The knock down of mTOR by expressing the specific shRNAs caused a substantial decrease in abundance of RanBP2 co-purified with mTOR antibody.

Our initial characterization of mTOR extracted from nuclei shows its co-elution with RanBP2 but not lamin B in the gel filtration fractions representing large complexes (Fig. 1D). This observation might also indicate the co-elution of mTOR and RanBP2 in a same protein complex. To examine whether a nuclear mTOR associates with RanBP2, we performed the nuclear localization and biochemical studies. Immunostaining of RanBP2 and mTOR has been performed by the previously validated antibodies [26, 27]. To assure a specificity of the nuclear mTOR detection by immunostaining, we have assessed the antibody. The mTOR antibody showed a high specificity, because detection of mTOR has diminished significantly in the mTOR knocked down cells and their nuclear enriched sub-cellular fraction (Suppl. Fig. 1). The immunostaining of mTOR in nuclei indicated that it is located primarily on the nuclear envelope facing a cytoplasm (Fig. 2A, C). We detected an uneven patched distribution of mTOR on a surface of the nuclear envelope and this staining pattern of mTOR has been similar to the staining of RanBP2 (Fig. 2B, C) demonstrating their strong co-localization (Fig. 2D) in a range of 80% (Table S1).

To study mTOR in the nuclear extract, we optimized immunoprecipitation of mTOR by increasing the stringency of the nuclear extraction buffer. We found that mTOR is co-purified with RanBP2 (Fig. 2E), and mTOR is detected in the pull down of RanBP2 (Fig. 2F). To address a specificity of the nuclear mTOR purification, we isolated mTOR from cells carrying a low level of mTOR attained by expression of the shRNAs targeting mTOR. The knock down of mTOR did not alter the expression level of RanBP2, but a low abundance of immunopurified mTOR protein from these cells showed only a small amount of RanBP2 co-purified with the mTOR antibody indicating that in our analysis co-purification of RanBP2 was mTOR-dependent (Fig. 2G). To further validate the mTOR and RanBP2 association, we isolated mTOR as the RanBP2 associating protein by pulling down RanBP2 with two additional antibodies (Suppl. Fig. 2A) and also by co-purification of RanBP2 by pulling down mTOR from two different human cancer cell lines MDA-MB-231 and HeLa (Suppl. Fig. 2B). In addition, within a set of immunoprecipitations from the nuclear extract including several NUP antibodies (NUP153, NUP62, NUP88) and also lamin B, only pull down of RanBP2 has been co-purified with mTOR (Suppl. Fig. 2C) suggesting that among nucleoporins RanBP2 is a main interacting component of mTOR.

Based on our immunostaining and biochemical studies, we identified that mTOR associates and is co-localized with the NPC component RanBP2 and resides within the nuclear envelope rim [28], the defined localization site of RanBP2. The distinct localization of the nuclear mTOR in a complex with RanBP2 might explain its salt dependent extraction representing a nuclear interface fraction. Thus, we identified that association of mTOR with RanBP2 represents the nuclear salt extractable complex of mTOR.

### The mTOR kinase activity independent of rapamycin and rictor regulates the nuclear abundance of ribosomal proteins and association of mTOR with RanBP2

RanBP2 as a component of NPC representing its major cytoplasmic filament has been identified as an important regulator of nuclear protein import [29, 30]. If mTOR by forming a complex with RanBP2 regulates its function, then inhibition of mTOR could interfere with the functional activity of RanBP2 and interrupt nuclear protein import.

**Figure 3:**
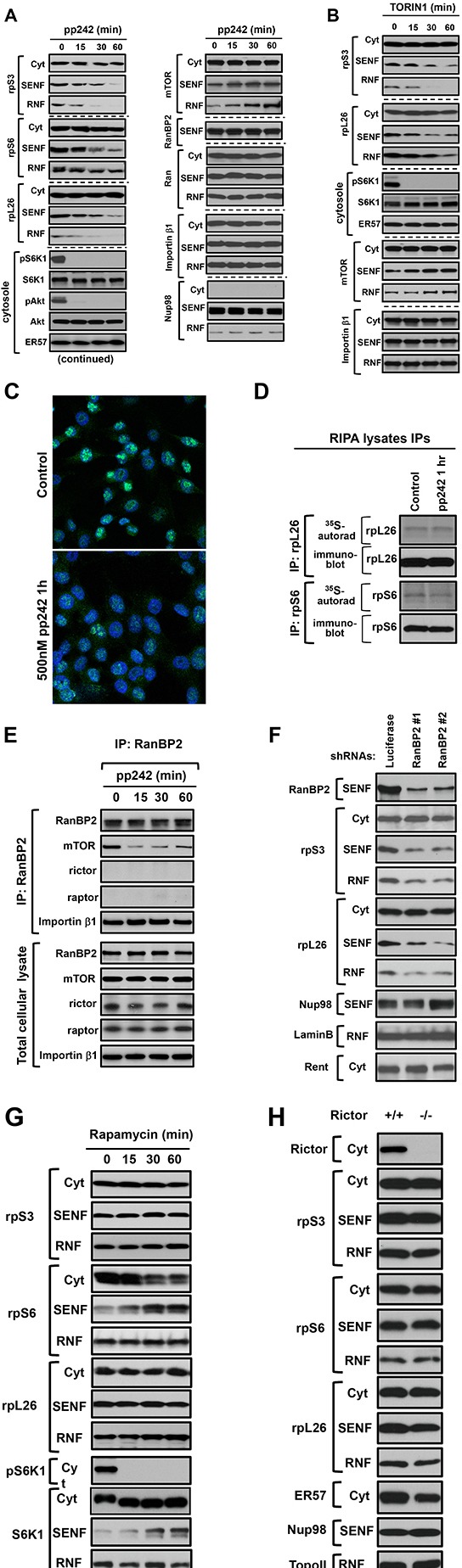
The mTOR kinase activity regulates the nuclear accumulation of ribosomal proteins independent of rapamycin and rictor expression (A-B) The nuclear abundance of ribosomal proteins is dependent on the mTOR kinase activity. MDA-MB-435 cells were incubated with 500 nM pp242 **(A)** or 100 nM Torin 1 **(B)** for the indicated time points and then lysed with the following fractionation to cytoplasmic (Cyt) and nuclear fractions (SENF and RNF). The isolated fractions were analyzed by immunoblotting for the levels of indicated proteins. The mTOR-dependent phosphorylation of S6K1 on Thr-389 and Akt on Ser-473 sites has been examined in the cytosolic fraction by the phospho-specific antibodies. Detection of the Nup98 and S6K1 proteins is shown as the loading controls. **(C)** Immunostaining of a free ribosomal protein L7a (rpL7a) in MDA-MB-435 cells incubated with or without 500 nM pp242 for 1 hour. The rpL7a antibody (Cell Signaling Technology, #2415) shows an exclusive nucleolar localization of rpL7a where this protein is enriched prior its assembly to ribosomal subunit. **(D)** Acute inhibition of the mTOR kinase activity does not suppress translation of the ribosomal proteins rpL26 and rpS6. MDA-MB-435 cells were incubated with 500 nM pp242 and labeled with ^35^S-methione/cysteine for 1 hr and cells were lysed in RIPA buffer. The rpL26 and rpS6 immunoprecipitates were analyzed by autoradiography and immunoblotting for the levels of newly synthesized proteins and total rpL26 and rpS6. **(E)** The kinase activity of mTOR is required for association of RanBP2 and mTOR. The actively growing HEK 293T cells were treated by 500 nM pp242 for the indicated times and were lysed in a buffer optimized to extract nuclear pore components (the buffer E). RanBP2 immunoprecipitates were prepared from the total cellular lysates and analyzed by immunoblotting. Abundance of the analyzed proteins in the total cellular lysate is shown in a lower panel. **(F)** The nuclear abundance of ribosomal proteins is dependent on RanBP2 expression. Following expression of the control (luciferase) or two different RanBP2 shRNAs, the MDA-MB-435 cells were analyzed by a sub-cellular fractionation and were analyzed by immunoblotting for the levels of indicated proteins as in A. **(G)** Rapamycin does not regulate the nuclear accumulation of ribosomal proteins, but induces rpS6 and S6K1translocation to the nuclear (SENF) fraction. MDA-MB-435 cells were incubated with 50 nM rapamycin for the indicated times. The sub-cellular fractions were isolated and analyzed by immunoblotting for the levels of indicated proteins as in A. **(H)** The nuclear accumulation of ribosomal proteins is rictor independent. Rictor null and wild type mouse embryonic fibroblasts (MEFs) were analyzed by a sub-cellular fractionation to the cytoplasmic and nuclear fractions. The obtained fractions were analyzed by immunoblotting with the indicated antibodies as in A.

The common biochemical methods of nuclei isolation by swelling cells in hypotonic buffer are not suitable for the nuclear protein transport studies because of a distortion of nuclei during purification by the simultaneous osmotic and mechanical forces that alter integrity of nuclei [22, 31]. To examine the mTOR-dependent nuclear proteins, we purified intact nuclei by the sub-cellular fractionation of cells in the isotonic buffer with a mild detergent as described in Figure 1. Initially, following a short-term inhibition of the mTOR kinase activity, we have examined several nuclear proteins and detected a decrease of the ribosomal proteins but not Nup98 [25], FBxO22 [32], topoisomerase II [33], EHD [34] in the nuclear fraction (Suppl. Fig. 3). To determine whether the nuclear accumulation of ribosomal proteins is dependent on mTOR, we analyzed abundance of the ribosomal proteins S3, S6, and L26 [35] in both nuclear (SENF and RNF) fractions following a potent inhibition of mTOR by pp242 [8] as detected by the mTOR-dependent phosphorylation of S6K1 and Akt (Fig. 3A, the lower panel). We observed a substantial decrease in abundance of the ribosomal proteins in the nuclear but not cytoplasmic fractions within 30 min of the mTOR inhibition, whereas a cellular distribution of the nuclear pore component Nup98 and the nuclear shuttling proteins Ran and importin β1 was not altered by a short-term inhibition of the mTOR kinase activity (Fig. 3A). An initial decline of the nuclear ribosomal proteins caused by pp242 was detected within 15 min implying that the nuclear shuttling of ribosomal proteins [15, 16, 18] is a dynamic process dependent on mTOR. Remarkably, a decline of the nuclear abundance of ribosomal proteins caused by inhibition of the mTOR kinase activity has carried an opposite effect by inducing accumulation of mTOR in the nuclear fraction (Fig. 3A, the right upper panel). Besides pp242, inhibition of the mTOR kinase activity by a different specific inhibitor Torin1 [36, 37] caused a similar effect on the nuclear abundance of ribosomal proteins and mTOR without affecting NUP98 or the nuclear shuttling proteins Ran and importin β1 (Fig. 3B). The data obtained by our fractionation study is supported by the immunostaining of a ribosome free ribosomal protein rpL7a that has been attained by the antibody recognizing rpL7a only prior its assembly into a ribosome subunit (Fig. 3C). Detection of a free rpL7a protein shows its predominant nuclear localization in actively growing cells (Fig. 3C, the upper panel) but following the treatment of cells with PP242 for one hour a nuclear staining of the ribosomal protein has been diminished substantially (Fig. 3C, the lower panel). Thus, our data indicate that the nuclear abundance of ribosomal proteins is dependent on the mTOR kinase activity.

It is known that the translation of mRNAs encoding ribosomal proteins is sensitive to inhibition of the mTOR kinase activity [38, 39]. Although the inhibitory effect on translation of ribosomal proteins by the mTOR kinase inhibitors might lead to a low abundance of ribosomal proteins in the nuclear fractions, the mRNA displacement from polysomes by pp242 has been reported to take place following the treatment of cells for 3 hrs [39]. It implies that the inhibitory effect on translation by the drug is not an immediate effect. In our study, we observed that inhibition of the mTOR kinase activity for 1 hr by pp242 did not suppress protein synthesis of the ribosomal proteins rpL26 and rpS6 as detected by the ^35^S-labeling experiment (Fig. 3D). This finding supports a functional role of mTOR in regulation of the nuclear shuttling rather than the translation of ribosomal proteins as an early immediate response to the drug. Alternatively, the mTOR kinase inhibitors could act by accelerating a degradation of ribosomal proteins known to take place at the nuclear site [40] by proteosomal pathway. We found that an effective inhibition of the proteosomal degradation by MG132, as detected by up-regulation of p27, did not alter the mTOR-dependent decrease of ribosomal proteins in the nuclear fraction (Suppl. Fig. 4). This observation excludes a role of the proteosomal degradation pathway of ribosomal proteins in action of the mTOR kinase inhibitors within 1 hr of treatment. Based on our data we hypothesize that a dynamic mTOR-dependent regulation of the nuclear abundance of ribosomal proteins is carried by a regulatory mechanism controlling the nuclear protein shuttling.

Our data indicate that mTOR regulates the nuclear accumulation of ribosomal proteins and it might carry this functional role in association with a critical mediator of nuclear import RanBP2. RanBP2 functions by docking of the nuclear shuttling proteins known as importins with their cargo at the nuclear import site. This docking step of importin β to RanBP2 filaments is essential for protein import and cell viability [41]. Based on the essential functional role of RanBP2 in mediating nuclear import, we hypothesized that association of mTOR with RanBP2 is a regulated interaction. To test our hypothesis, we examined the RanBP2 complex by immunoprecipitation of RanBP2 form total cellular lysates. We obtained total cellular lysates by using the optimized buffer capable of extracting RanBP2 and preserving association of mTOR with RanBP2 (Fig. 3E). Coherent with our initial observation, the nuclear pore component RanBP2 was co-purified with mTOR but not with the known mTOR interacting proteins rictor or raptor indicating that, most likely, mTOR in association with RanBP2 forms a complex distinct from mTORC1 and mTORC2. We found that inhibition of the mTOR kinase activity by pp242 for up to 1 hr did not alter the levels of RanBP2, mTOR, importins β, or ribosomal proteins in the total cellular lysates, but the association of RanBP2 with mTOR was sensitive to inhibition of the mTOR kinase activity (Fig. 3E and Suppl. Fig. 5). In addition, the knock down of RanBP2 also caused a low accumulation of ribosomal proteins in the nuclear fractions (Fig. 3F) that resembles effect of the mTOR kinase inhibition (Fig. 3A, B). These findings suggest that mTOR in association with RanBP2 regulates the nuclear accumulation of ribosomal proteins.

If mTOR forms a distinct complex with RanBP2 within a nuclear interface, it might function independent of mTORC1 or mTORC2. Indeed, we found that inhibition of mTORC1 by its specific inhibitor rapamycin for 1 hr did not induce any reduction of rpS3, rpS6, or rpL26 in the nuclear fractions (Fig. 3G). Rapamycin was effective in inhibition of mTORC1 as detected by phosphorylation of S6K1 [19] but it induced an opposite translocation effect of rpS6 and S6K1 from the cytoplasmic to the nuclear fraction (Fig. 3G). This effect is related to a specific inhibition of mTORC1 by rapamycin that is distinct from the effects caused by inhibition of the mTOR kinase activity. We also examined the mTORC2 deficient cells caused by a loss of rictor [42]. The nuclear extracts obtained from the rictor null cells did not show any change in a nuclear abundance of the ribosomal proteins (Fig. 3H). Our data show that an acute inhibition of mTORC1 by rapamycin or deficiency of mTORC2 does not interfere with the nuclear accumulation of ribosomal proteins.

### The mTOR kinase activity regulates the nuclear import of ribosomal proteins

**Figure 4:**
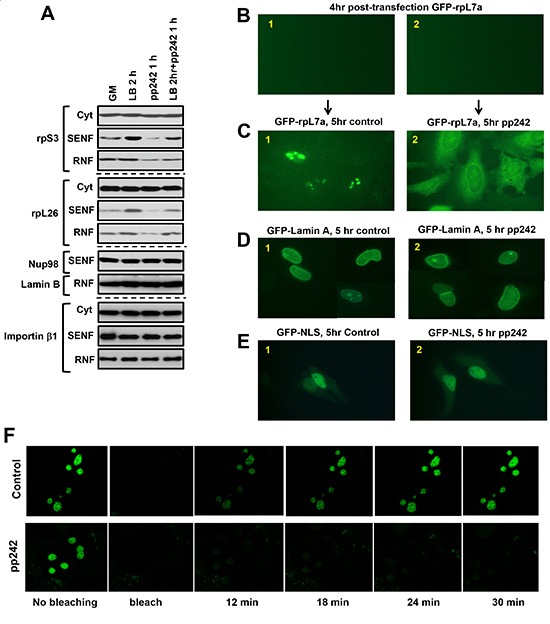
Nuclear import of the GFP-tagged ribosomal protein rpL7a is dependent on the mTOR kinase activity **(A)** The nuclear accumulation of ribosomal proteins by inhibition of the nuclear export is dependent on the mTOR kinase activity. To interfere with the nuclear export MDA-MB-435 cells were incubated with 200 nM leptomycin B for 2 hours. The leptomycin B treated cells were treated with or without pp242 at concentration 500 nM for 1 hour. The cytosolic and nuclear abundance of rpS3, rpS6, and rpL26 has been analyzed by immunoblotting of the nuclear fractions SENF and RNF. **B-E**, HeLa cells were transiently transfected with GFP-rpL7a, GFP-Lamin A, or GFP-tagged with the nuclear localization signal (GFP-NLS). Localization of the GFP-tagged proteins has been detected by fluorescent microscopy. The following images are presented: **(B1-B2)** after four hours of transfection, expression of GFP-L7a at the initial time point is not detectable, **(C1)** Control cells shows accumulation of GFP-RPL7a in nucleolus, **(C2)** Cells incubated with 1 μM of pp242 shows accumulation of GFP-L7a mostly in cytoplasm after 5 hours of treatment, **(D1-D2** and **E1-E2)** The nuclear import of lamin A or GFP-NLS is not sensitive to the treatment of cells with 1 μM pp242. Both proteins show nuclear staining in cells with or without the drug treatement. **(F)** The nuclear import of GFP-rpL7a is dependent on the mTOR kinase activity. HeLa cells were transiently transfected with the expression plasmid encoding GFP-rpL7a protein and incubated with (the lower panel) or without (the upper panel) 1 μM of pp242. Following the photo-bleaching by a laser pulse, the recovery of rpL7a in nucleolus has been monitored by fluorescent microscopy. The images were taken within 6 min intervals.

RanBP2 has been identified as a critical regulator of nuclear import. mTOR by association with RanBP2 might play a role in regulation of the nuclear import of ribosomal proteins because their nuclear abundance is sensitive to inhibition of the mTOR kinase activity. To dissect the functional role of mTOR associated with RanBP2 in the nuclear import of ribosomal proteins, we examined the nuclear accumulation of ribosomal proteins following an obstruction of nuclear export by leptomycin B [43]. Within two hours of treatment of cells with leptomycin B, we observed a higher accumulation of rpS3 and rpL26 in the nuclear fraction indicating an effective blocking of nuclear export by the drug without affecting abundance of importin β (Fig. 4A). Inhibition of the mTOR kinase activity carried an opposite effect as detected by a decreased abundance of the ribosomal proteins. We found that the treatment of cells with leptomycin B for 2 hr and pp242 for 1 hr did not induce an accumulation of the ribosomal proteins to the level detected by the treatment of cell with leptomycin B alone. This experiment suggests that inhibition of the mTOR kinase activity diminished the effect of leptomycin B by restraining the nuclear import of ribosomal proteins.

To further pursue the mTOR-dependent nuclear import of ribosomal proteins, we analyzed a sub-cellular localization of the transiently expressed GFP-tagged rpL7a [18] or lamin A [44] protein in live cells. It is known that the GFP-tagging of most of ribosomal proteins including rpL7a prevents their assembly into ribosomes and these proteins are actively degraded at the nuclear site [18, 40]. The rpL7a protein has been selected in our nuclear import study, because the GFP tagged rpL7a gets translocated to the nucleolar site and degraded within nucleus. We assumed that inhibition of the nuclear import of GFP-rpL7a would result in a cytosolic accumulation of this ribosomal protein by preventing its nucleolar localization. An initial appearance of the GFP-tagged protein expression has been detected after 4 hrs of transfection (Fig. 4B). At this time point, we inhibited the mTOR kinase activity by pp242 and monitored localization of the GFP-tagged proteins. As previously reported [45], we detected accumulation of the GFP-tagged rpL7a in nucleolus (Fig. 4C, panel 1). Inhibition of the mTOR kinase activity did not suppress expression of the GFP-rpL7a protein as indicated by detection of the recombinant protein (Suppl. Fig. 6). Following inhibition of the mTOR kinase by pp242 for 5 hrs, we observed a robust expression of GFP-rpL7a in cytoplasm but not in nucleolus (Fig. 4C, panel 2), whereas control cells show accumulation of this ribosomal protein only in nucleolus (Fig. 4C, panel 1) as reported previously [18, 45]. Most likely, a high accumulation of rpL7a in cytoplasm induced by inhibition of the mTOR kinase activity is caused by a separation of the ribosomal protein from its degradation site known to be taking place in nucleus [40]. In the same setting inhibition of mTORC1 by rapamycin did not show any effect on the localization of GFP-rpL7a in nucleolus (data not shown). Importantly, inhibition of the mTOR kinase activity did not interfere with the nuclear localization of the GFP-tagged lamin A protein (Fig. 4D) or the NLS containing GFP protein (Fig. 4E) indicating a selective role of mTOR in regulation of the nuclear import of ribosomal proteins. We also addressed a role of the mTOR kinase in regulation of the nuclear import by the photo-bleaching experiment (Fig. 4F). Following a transient expression of the GFP-rpL7a protein in HeLa cells, we detected accumulation of this protein in nucleolus [18]. The laser pulse was effective in bleaching the fluorescent recombinant protein at the nucleolar site. The time-lapse analysis indicated a dynamic nuclear import of the ribosomal protein, because we detected an initial recovery the GFP-rpL7a protein signal at the same nucleolar location after 12 min and its full recovery after 24 min following the photo-bleaching (Fig. 4F, the upper panels). Importantly, the treatment of cells with the mTOR kinase inhibitor pp242 prior to the photo-bleaching has prevented a recovery of the GFP-rpL7a protein at a nucleolar site (Fig. 4F, the lower panels). Our study indicates that the kinase activity of mTOR plays a role in regulation of a dynamic nuclear import of ribosomal proteins. The mTOR-dependent regulation of the nuclear import is an early immediate response to inhibition of the mTOR kinase activity. Inhibition of mTOR is known to suppress ribosomal mRNA transcription [12] and within 1 hr time frame we did not detect a substantial effect on the ribosomal RNA expression (Suppl. Fig. 7). It supports our observation that the nuclear import regulation is an early immediate response to inhibition of the mTOR kinase activity.

## DISCUSSION

Nuclear transport is a selective process of translocation of macromolecules by NPC across nuclear envelope. Each NPC represents a transport channel by assembling multiple copies of approximately 30 components, known as nucleoporins. A permeability barrier and receptor mediated traffic of NPC is mediated by nucleoporins lining the central channel and containing phenylalanine-glycine (FG) repeats [46]. While functional studies of NPC with the focus on its structural aspects have been actively pursued for years [47, 48], regulation of the NPC mediated nuclear transport remains poorly characterized. Here, we describe the mTOR-dependent nuclear import of ribosomal proteins.

In our study, we show that the nuclear import of ribosomal proteins is a highly regulated process, where mTOR in association with the nuclear pore component RanBP2 controls the nuclear import of ribosomal proteins. RanBP2 has been defined as a critical mediator of nuclear import and this vital role of RanBP2 is conducted via docking of importin β to its N-terminal fragment containing the NPC-binding domain, three FG repeats, and Ran Binding Domain (RBD) [41]. It has been shown that importin β shuttling proteins carry an active nuclear import of ribosomal proteins without involvement of their interacting importin α partners known to function as the NLS receptors [15, 16, 49]. Our study suggests that mTOR in association with RanBP2 regulates the nuclear import by controlling a shuttling activity of importin β proteins accountable for import of ribosomal proteins. This regulation requires the kinase activity of mTOR implying a critical role of phosphorylation in this process. The mTOR-dependent phosphorylation of RanBP2 or imporitn β proteins and other players of nuclear import associated with RanBP2 [30, 41] might steer a regulation of the nuclear import of ribosomal proteins. Our initial characterization of this process does not show the mTOR-dependent changes of docking importin β1 to RanBP2, although inhibition of the mTOR kinase activity was causing a disassociation of RanBP2 and mTOR (Fig. 3E and Suppl. Fig. 5). It is possible that docking of other importin β2, 5, and 7 proteins reported to carry the nuclear import of ribosomal proteins [15] is dependent on mTOR, but in our studies we detected only weak association of these shuttling proteins with RanBP2 (data not shown). Alternatively, mTOR might facilitate the nuclear import by an enrichment of ribosomal proteins at the cargo-docking site of NPC located in proximity of RanBP2 [30, 41]. This type of regulation could explain why mTOR promotes the nuclear import of ribosomal proteins without altering the NLS-dependent import of nuclear proteins.

A distinct localization of RanBP2 suggests that mTOR associates with RanBP2 on the cytosolic nuclear interface that is coherent with the co-localization data (Fig. 2A-D). This localization is determined by the functional role of mTOR in regulation of the nuclear import that is different from the reported cytoplasmic localization sites of mTORC1 and mTORC2 [10, 27] that indicates a novel functional role of mTOR independent of its defined complexes mTORC1 and mTORC2. It has been shown previously that mTOR can exert its effects largely independent of mTORC1 and mTORC2 [50, 51], and we believe it is also the case in our study. The nutrient dependent translocation of mTORC1 on lysosomal surface has been shown to be critical in regulation of mTORC1 [27]. In our study we found that inhibition of the mTOR kinase activity by pp242 or Torin1 induced a translocation of mTOR to the nuclear fraction. Most likely, the drug has provoked a translocation of mTOR to nuclear interface as a compensatory mechanism to deterrence of the kinase dependent association of mTOR with RanBP2. A dynamic nuclear translocation of mTOR induced by its kinase inhibition might indicate the vital role of mTOR on nuclear envelope related to regulation of nuclear import or other unknown functions of mTOR.

In our study, the biochemical characterization of mTOR led to discovery of its novel localization site within nuclear envelope. In the previous studies, purification of the mTOR complexes has been carried in a mild lysis buffer [19, 20] that is effective for extraction of the cytoplasmic but not nuclear envelope proteins (data not shown). It might explain why isolation of the nuclear envelope mTOR complex has remained elusive. We find that a sub-cellular fractionation by excluding the cytosolic fraction and analysis of the purified intact nuclei is an effective approach to extract the nuclear envelope mTOR complex. Under these settings, mTOR and its interacting proteins rictor and raptor are abundant in the cytosolic fraction and only a substantial level of mTOR is detected in the nuclear fraction indicating that mTORC1 and mTORC2 are predominantly located in cytoplasm but not in nucleus. In contrast, in the previous study both mTOR complexes have been detected in the nuclear fraction [52]. This disagreement can be explained by the difference in isolation of nuclei. We carried purification of the intact nuclei under isotonic as opposed to the commonly used hypotonic lysis condition. Our initial application of the hypotonic lysis conditions also indicated a presence rictor and raptor in the nuclear fraction (data not shown). Although the hypotonic buffer is effective in rupturing the plasma membrane, it also results in expansion of nuclei and subsequently in altering the integrity of NPCs and causing a disruption of the nuclear/cytoplasmic barrier [22, 31]. This disruption has been a major setback for biochemical studies of nuclear dynamics. The nuclei purified under isotonic buffer conditions retain their integrity and this approach excluding osmotic stress of nuclei allows biochemical studies of nuclear dynamics. We found that inhibition of the mTOR kinase activity by pp242 or Torin1 within 30 min caused a substantial decrease in abundance of ribosomal proteins in the nuclear but not cytoplasmic fraction. The cell-based assays further indicated that mTOR controls the nuclear abundance of ribosomal proteins by regulating the nuclear import. The fact that the nuclear abundance of ribosomal proteins was not affected by inhibition of mTORC1 by rapamycin or loss of mTORC2 suggests that mTOR carries its role in regulation of the nuclear import in association with RanBP2 but not as a component of mTORC1 or mTORC2. Remarkably, compared to the mTOR kinase inhibitors, rapamycin induced the opposite effect on the ribosomal protein rpS6 and its kinase S6K1 by inducing their nuclear accumulation (Fig. 3G). It is unlikely, that this effect is related to the mTOR-dependent phosphorylation of S6K1 and rpS6, because similar dephosphorylation of both proteins occurs by incubation of cells with the mTOR kinase inhibitor pp242. This effect might be linked to a partial inhibition of mTORC1 by rapamycin and enhancing the localization of rpS6 and S6K1 within a nuclear interface.

In accordance with the previous studies [15, 16], our data demonstrate that the nuclear import of ribosomal proteins is a dynamic process. We show that this process requires the mTOR kinase activity. A decreased nuclear abundance of ribosomal proteins caused by inhibition of the mTOR kinase activity is detected within 30 min, whereas the mTOR-dependent recovery of the ribosomal protein at the nucleolar site has been observed within 18 min following photo-bleaching. These data indicate that a dynamic nuclear import of ribosomal proteins is regulated by the mTOR kinase activity. On the other hand, the mTOR kinase inhibitors are known to be effective in obstruction of protein synthesis including the translation of ribosomal proteins encoded by mRNAs containing 5′ terminal oligopyrimidine (TOP) motifs [38, 39]. It has been reported that treatment of cell with pp242 displaces ribosomal RNAs from polysomes following 3 hrs of treatment [39] and our ^35^S-labeling experiment did not show a substantial effect on protein synthesis of rpL26 and rpS6 within 1 hour of treatment of cells with pp242 (Fig. 3D). Based on these observations, we propose that the mTOR-dependent effect on the nuclear abundance of ribosomal proteins within the first hour of inhibition is mostly related to obstruction of the nuclear import, whereas the inhibitory effect on protein synthesis of ribosomal proteins takes place following the treatment of cells for 2 hours by Torin1 or for 3 hours by pp242 [38, 39].

Coordinated assembly of ribosomes takes place at the distinct nuclear site known as nucleolus [53]. It indicates that nuclear transport plays a role in ribosomal biogenesis. It assumes that ribosomal biogenesis depends not only on a rate of synthesis of ribosomal components but also on rates of the nuclear import of ribosomal proteins and nuclear export of assembled pre-ribosomes to cytoplasm. CRM1 is known to mediate the nuclear export of ribosomes [18], whereas several importin β proteins have been reported to mediate an active nuclear import of ribosomal proteins [15]. Regulation of the nuclear import of ribosomal proteins has not been addressed previously. Our data indicate that mTOR by association with RanBP2 regulates the nuclear import of ribosomal proteins. We propose that this functional role of mTOR contributes to regulation of ribosomal biogenesis by promoting translocation of ribosomal proteins to the site of ribosome assembly. It implies that the mTOR-dependent nuclear import of ribosomal proteins regulates a rate of ribosomal biogenesis. Ribosomal biogenesis is a hallmark of cell growth and activation of this process is associated with tumorigenesis [12, 54]. To support abnormal cell growth, cellular transformation is impelled by at least seven-fold increase in a rate of the nuclear protein import [55]. A high expression of mTOR is common in human cancers [56–59] and an elevated nuclear dynamics in cancer cells might be related to deregulation of mTOR. It is known that mTOR as a central component of mTORC1 and mTORC2 drives cell growth and proliferation [3]. Besides the functional roles of mTORC1 and mTORC2, our study describes a novel function of mTOR that might be critical for a high growth rate of cancer cells. Overall, we show that mTOR in association with RanBP2 controls a dynamic flow of ribosomal proteins to nuclear compartment.

## MATERIAL AND METHODS

### Materials and cell culture

MDA-MB-435, HEK 293T, A549, LNCaP, HeLa, MDA-MB-231 cells were obtained from American Type Culture Collection. Pre-packed Sephacryl S-500 column was purchased from HE Healthcare (Piscataway, NJ, USA). Reagents were obtained from the following sources: DMEM/F12 from Life Technologies, Fetal Bovine Serum (FBS) from Hyclone, Fugene 6 transfection reagent and the complete protease inhibitor cocktail from Roche (Indianapolis, IN, USA). Leptomycin B was purchased from LC labs, pp242 from Chemdea (Ridgewood, NJ, USA). Three RanBP2 (sc-7418; sc-28577; sc-15442), two Nup62 (sc-1916; sc-25523), two Importin β (sc-137016; sc-1863), Nup153 (sc-20590), Nup88 (sc-13609), Ran (sc-271376), mTOR (sc-1549), Lamin B (sc-6216), TopoIIα (sc-5348), rpL28 (sc-14151), Rent1 (sc-48802), EHD (sc-32917), p53 (sc-6243), α-Tubulin (sc-8035), HRP-labeled anti-rabbit/anti-mouse/anti-goat secondary antibodies were purchased from Santa Cruz Biotechnology (Santa Cruz, CA, USA). Nup98 (2598), two rpL7a (2415; 2403), rpS6 (2317), rpS3 (9538), pS6K1 (9234), pS6 (2211), mTOR (2983; 4517), Rictor (2114), Raptor (2280), Akt (4685), pAkt (4058) were purchased from Cell Signaling Technology (Danvers, MA, USA). Nup88 (611896) was purchased from BD Biosciences (San Jose, CA, USA). RanBP2 (ab64276) was purchased from Abcam (Cambridge, MA, USA). RanBP2 (A301-797), Nup153 (A301–788A), two rpL26 (A300-686A; A300–685A), two rpS6 (A300-556A; A300-557A), mTOR (A301-142A) were purchased from Bethyl Laboratories (Montgomery, TX, USA). Alexa-coupled anti-mouse/anti-rabbit secondary antibodies were obtained from Invitrogen (Carlsbad, CA, USA). Buffers: (1) Buffer A for size exclusion chromatography: 40 mM Hepes pH 7.5, 160 mM KCl, 1 mM MgCl_2_ (2) Buffer B for cellular fractionation: 40 mM Hepes pH 7.5, 160 mM KCl, 2.5 mM EGTA, 2.5 mM EDTA, 0.5% Glycerol, 0.5% NP-40. (3) Buffer C for size exclusion chromatography and cellular fractionation: Buffer B with 0.1 M LiCl added fresh. (4) Buffer D for obtaining SENF fractions: Buffer B with 0.2 M LiCl added fresh. (5) Buffer E for immunoprecipitations: 5 mM Tris-HCl pH 7.4, 0.5% Triton X-100, 0.5% Deoxycholate, 2.5 mM MgCl_2_, 1.5 mM KCl with 0.2 M LiCl added fresh. The buffer E was applied to lyse the intact nuclei or cells as described in the figure legends to obtain lysates for immunoprecipitations. All buffers prior to use were supplemented with the protease inhibitor cocktail from Roche and phosphatase inhibitors (560 nM Okadaic acid and 50 nM calyculin A).

### Gel-filtration chromatography by Sephacryl S-500 column

The cellular lysates for the size exclusion chromatography were prepared in Buffer A under freeze/thaw lysis cycle conditions, or after the lysis in Buffer B with following nuclear fractionation in Buffer C. About 15 mg of extracts were applied to gel filtration analysis and eluted at a flow rate of 0.5 mg/ml with the same sample buffer (Buffer A or Buffer C, respectively). Each fraction was analyzed by immunoblotting using appropriate antibodies. The Sephacryl S-500 column was selected to minimize disintegration effect on complexes during fractionation because it is packed with the resin that has large size pores with a mean exclusion size of 200 nM and effective in separation of massive macromolecules or complexes at low pressure [60, 61]. To preserve native mTOR complexes, cells were lysed in Buffer A without detergent by freeze-thaw method and eluted with the same buffer. The nuclear mTOR complex in SENF fraction was analyzed by gel-filtration chromatography by running the nuclear fraction through Sephacryl S-500 column. About 15 mg of the nuclear extracts prepared from MDA-MB-435 cells were subjected to gel filtration analysis and eluted at a flow rate of 0.5 mg/ml.

### Isolation of the nuclear fractionations

Intact nuclei were isolated in the isotonic buffer containing a low detergent concentration without applying osmotic stress and mechanical force by optimizing the method of isolation of intact nuclei from hematopoietic cells [62]. Thirteen million of MDA-MB-435 cells were grown overnight in 145 mm dished. Following rinsing of cells with cold PBS, the cells were lysed in 0.3 ml of the Buffer B by collecting lysate into 1.5 ml tubes and rotating for 30 min at 4°C. Nuclei were pelleted by centrifugation at 500 g for 5 min. The pellet of nuclei was gently washed with Buffer B without NP40 detergent, centrifuged at 500 g for 5 min and the supernatant was discarded. The purified nuclei were further lysed in the Buffer D for 40 min and the salt extractible nuclear fraction (SENF) was obtained as the supernatant by centrifugation at 14000 g for 10 min. The nuclear pellets were dissolved in 8M Urea buffer to obtain the Residual Nuclear Fractions (RNFs). The two sequential steps of nuclear sub-fractionation have been applied only in the initial study (Figure 1) were a low salt concentration (SENF-0.1 M LiCl) step was followed by a high salt concentration (SENF-0.2 M LiCl). To isolate SENF-0.1 M LiCl, the nuclei were incubated in Buffer C containing 0.1 M LiCl for 1 hour at 4°C and separated by centrifugation at 500 g. Then the pellet was incubated in Buffer D containing 0.2 M LiCl for 1 hour to isolate SENF-0.2 M LiCl fraction and separated by centrifugation at 500 g. This two step nuclei sub-fractionation technique was used to demonstrate efficiency of the protein extraction from nuclei without breaking the nuclear structure. In the following studies the isolation of SENF was performed by extracting nuclei in the buffer D containing 0.2 M LiCl.

### Immunostaining and confocal microscopy

mTOR, as a central component of mTORC1 and mTORC2, is an abundant protein in the cytoplasm. Due to that fact we optimized a cell fixation technique to emphasize immunostaining of the nuclear mTOR. In order to show the nuclear mTOR, cells were fixed in a Methanol-based fixative that does not crosslink proteins as compared to the Aldehyde-based fixatives [63]. By testing different fixation solutions we found that a cold Methanol/Ethanol mixture of 1:1 ratio removes most of the cytoplasmic and retains the nuclear mTOR. After that cells were fixed with paraformaldehyde. For specific immuno-staining of mTOR and RanBP2 we applied previously validated antibodies [26, 27]. Fifty thousand cells per well were grown in 4 well glass chamber for 24 hr at 37°C with 5% CO_2_. Cultured cells were washed with PBS and fixed immediately with a −20°C Methanol/Ethanol mixture (1:1). After three washes with PBS, the cells were permeabilized with 0.2% Triton X-100 for 15 min, then fixed with 4% paraformaldehyde. After another PBS wash, the cells were blocked with 5% BSA for 30 min. and incubated with mTOR-specific and RanBP2-specific antibodies. The labeled proteins were detected using the appropriate Alexa 488 or Alexa 594-conjugated secondary antibodies for 1 hour at room temperature. Images obtained with confocal laser scanning microscope (LSM710, Zeiss, Jena, Germany) (x63) were analyzed using ZEN 2011 software that calculated Pearson's correlation coefficient and overlap coefficient, according to Manders.

### Immunoprecipitation and immunoblotting

The cellular lysates or cellular fractions were incubated with the appropriate antibodies for 3 hours or overnight at 4°C and then pulled down with G-protein agarose beads following 90 min of incubation. RanBP2 was pulled down with the following RanBP2 antibodies: Santa Cruz (sc-28577; sc-15442), Bethyl Labs (A301-797), and Abcam (ab64276). The mTOR antibody from Santa Cruz (sc-1549) was used to pull down mTOR. In order to improve nuclear protein extraction for immunoprecipitation, the extracts were obtained by adding 0.5% Deoxycholate and LiCl (Buffer E). The G-protein beads were washed four times with the lysis buffer and the imunopurified proteins were analyzed by immunoblotting by boiling the beads in the SDS sample loading buffer. Co-purified proteins were detected by the indicated antibodies. The ^35^S-labeling experiment was performed as described previously [64]. MDA-MB-435 cells (4 × 10^6^) growing in 100 mm dishes were rinsed once in methionine- and cysteine-free DMEM, and then incubated in 3.5 ml of the same medium containing 10% dialyzed serum and 0.1 mCi/ml of ^35^S-methionine/^35^S-cysteine (Express Protein Labeling Mix, Perkin Elmer). At the same time cells were treated with 500nM PP242 or vehicle control for 1 hr min. After allowing the cells to label and treated with pp242 for 1 hr, the cells were washed twice with cold PBS and lysed in a RIPA buffer. The total cellular lysates were applied for immunoprecipitations as described above.

### Lentiviral vector, lentivirus production and infection

Lentiviral production has been performed, as described previously [65]. Plasmids were propagated in and purified from XL-10 Gold bacterial cells. One day prior to transfection, Human Embryonic Kidney (HEK) 293T cells (1.2 × 10^6^) were plated on 6 cm plates in 3 ml DMEM/F12 supplemented with 10% fetal bovine serum. For production of lentiviruses, the HEK 293T cells were transfected with 1 μg of transfer vector p-LOX1, 1 μg of envelop coding plasmid CMV VSV-G, and 2 μg of Δ-VPR, using Fugene 6 transfection reagents, according to either the manufacturer's instructions or the calcium phosphate transfection method. Lentiviruses were harvested 48 hours after the transfection. One day prior to infection, cells to be infected were seeded in six-well dishes. The viral supernatant was added to the culture medium at a ratio of 1:1 in the presence of polybrene (8 μg/ml) and the cells were spun at 1200 g for 45 min at 32°C in order to increase the infection efficiency. In the following 24 hours the cells were incubated with retroviruses and the infected cells were passaged and selected with puromycin (3.4 mg/ml for 2 days). The cells were then used for the immunoprecipitation assay followed by immunobloting. To knock down RanBP2 we used pGIPZ shRNAmir lentiviral vector plasmids clones V3LHS_365487 and V3LHS_365488 (Thermo Scientific, Open Biosystem, Huntsville, AL, USA), by a similar approach described above.

### The nuclear import assay

To show the role of mTOR in the nuclear import of ribosomal proteins in live cells, we developed the *in vivo* import assay by expressing the GFP-tagged ribosomal protein rpL7a (GFP-rpL7a) [45] and Lamin A (GFP-Lamin A) [44]. We proposed that inhibition of the nuclear ribosomal import at the initial stage of the GFP-rpL7a expression would prevent its translocation to nuclei of live cells. An initial signal of the GFP-tagged protein expression was detected at 4 hrs following transfection and at this time the mTOR kinase activity has been inhibited by pp242. HeLa cells were grown on 35 mm glass bottom dishes and transfected with GFP-rpL7a in a vector pCMV6-AC-GFP (Origene, Rockville, MD, USA) or pBABE-puro-GFP-Lamin A (Addgene, Cambridge, USA). 4 hours post-transfection, the mTOR inhibitor has been added every two hours. To detect a sub-cellular localization of GFP-tagged proteins following inhibition of mTOR for 5 hr and the fluorescent microscope images of live cells were taken at this time point.

### Photo-bleaching study

One day prior to GFP-L7a transfection, 100,000 HeLa cells were seeded in polylysine-coated 35 mm glass bottom dishes (Mattek). On the next day, the cells were transiently transfected with 0.5 μg GFP-L7a plasmid and Lipofectamin (Lipofectamine: plasmid DNA ratio of 3:1). Transfected cells after 24 hrs were treated with 500 nM pp242 for 1 hr. 30 min, and additional 1 μM of pp242 was added before photo-bleaching experiment. Prior to bleaching, images of cells were collected using 2% of total laser power with excitation at 488 nm, scanning an area of 150 μm^2^ at a rate of 12 μs/pixel. Nuclear bleaching of control and pp242-treated cells was performed in an area covering approximately 80 μm^2^, which contained six to eight nucleolus, at a rate of 50 μs/pixel, by applying 80% of the laser power twice. After photo-bleaching, the cells were immediately scanned and the recovery of fluorescence was monitored by acquiring subsequent images at 6 min intervals for up to 30 min.

### RNA extraction and Quantitative Real time PCR Analysis

MDA-MB-435 cells were grown in complete culture medium until 60-70% confluence. Cells were treated with 500 nM of pp242 for 1h. Total cellular RNA was isolated using RNeasy Mini Kit (QIAGEN, Hilden, Germany) according to the manufacturer's instructions. To remove any residual genomic DNA, RNA samples were treated with DNase (QIAGEN). Quantitative real-time PCR analysis was performed according to the described protocol [66]. The primers and probes for 18S and GAPDH were obtained from Applied Biosystems (Carlsbad, CA, USA).

## SUPPLEMENTARY FIGURES AND TABLE


