# Supplementary material for: The nuclear import of ribosomal proteins is regulated by mTOR
Source: Oncotarget. 2014 Oct 3;5(20):9577–93. doi: 10.18632/oncotarget.2473 (PMC4259421; doi:10.18632/oncotarget.2473)
Supplement: Supplementary file 1 [file oncotarget-05-9577-s001.pdf]

SUPPLEMENTARY FIGURES AND TABLE

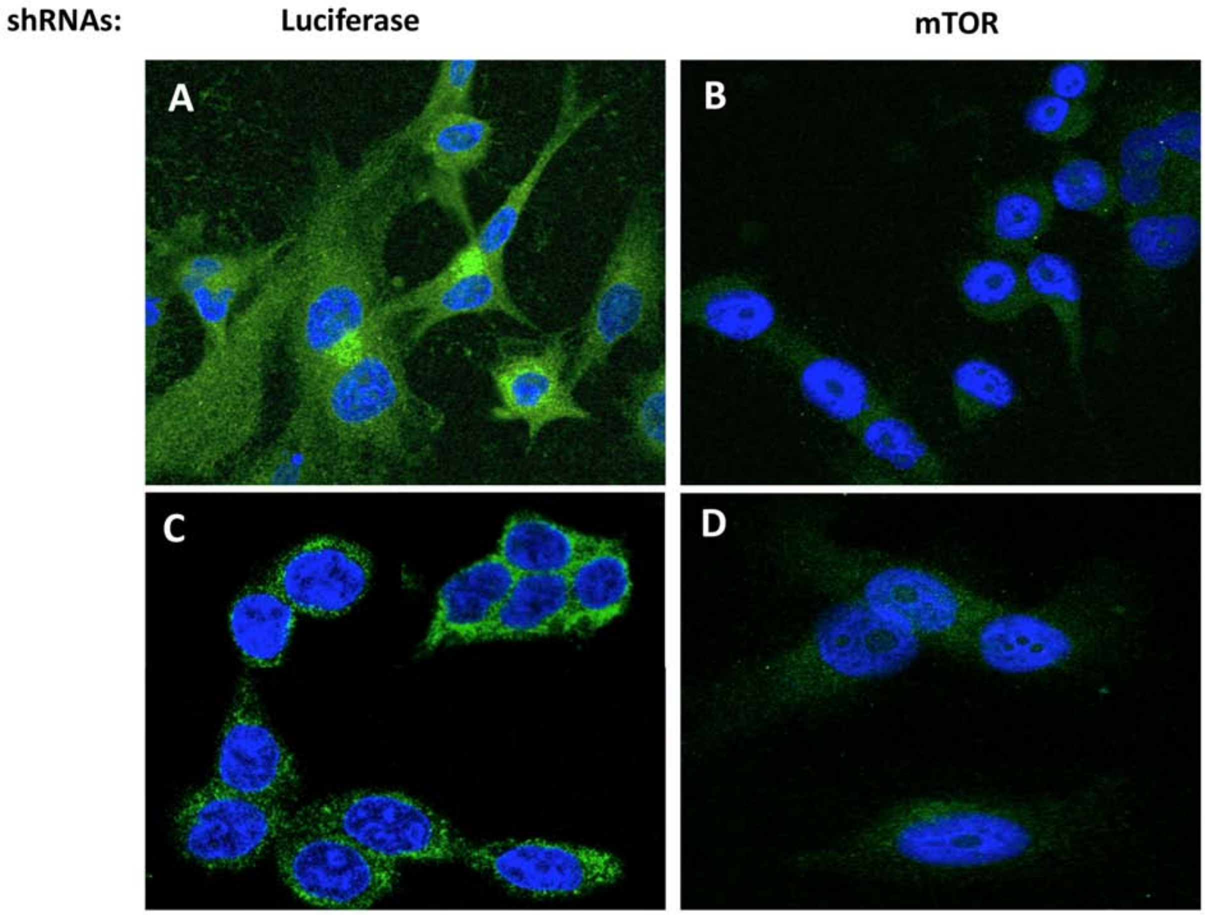

Supplementary Figure S1: Immunostaining of mTOR is specific. Validation of the mTOR antibodies has been perfomed by immunostaining of the mTOR knock down in HeLa cells. A-B, Immunostaining of mTOR in cells expressing non-specific luciferase **(A)** or mTOR **(B)** shRNAs. C-D, Immunostaining of mTOR in the nuclear enriched fraction from cells expressing non-specific luciferase **(C)** or mTOR **(D)** shRNAs. As described in the methods section for cellular staining cells were fixed by formaldehyde, but for the nuclear enrichment cells were initially fixed by a methanol/ethanol solution with the following formaldehyde fixation.

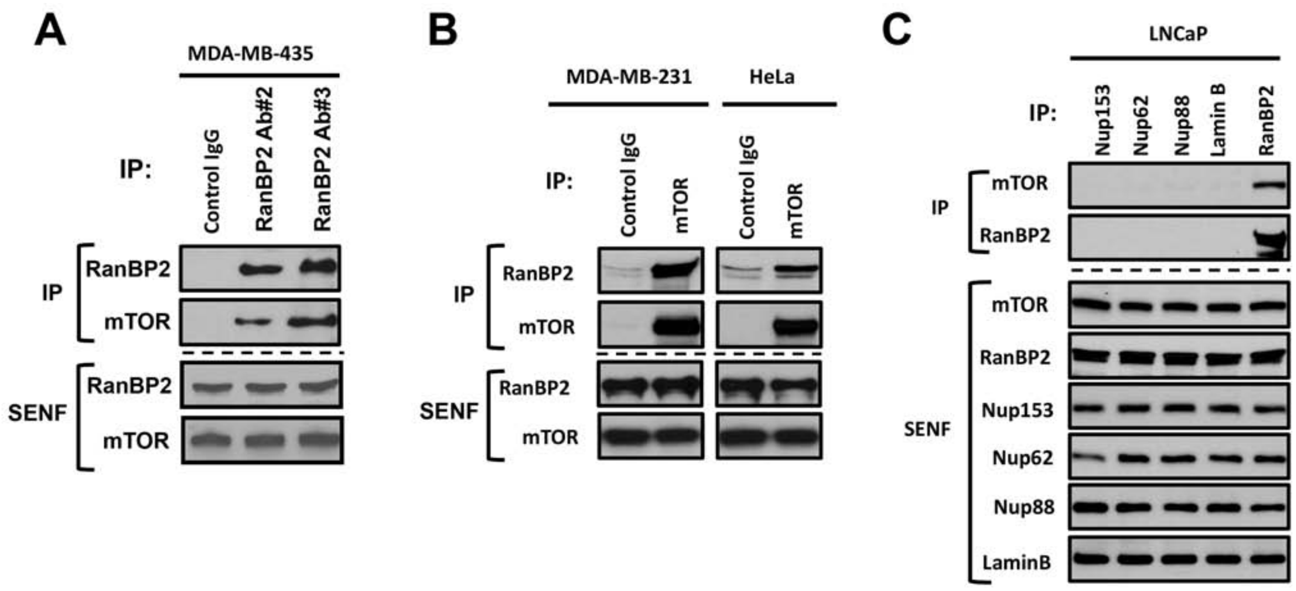

Supplementary Figure S2: Association of mTOR with RanBP2. The immunoprecipitations of RanBP2 or mTOR and also different NPC components from the salt extractable nuclear fraction (SENF) obtained from different human cancer cell lines have been analyzed by immunoblotting with the indicated antibodies. **(A)** RanBP2 is co-purified with mTOR from the SENF extract of MDA-MB-435 cells by two additional RanBP2 antibodies. **(B)** The pull down of mTOR from SENF is co-purified with RanBP2 in other two cell lines (MDA-MB-231 and HeLa) **(C)** Analysis of immunoprecipitates of the different NPC components and lamin B from SENF of LNCaP cells.

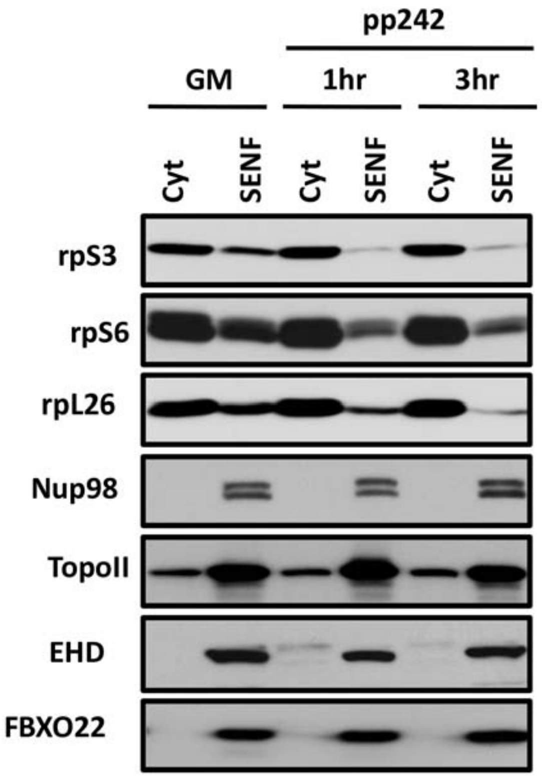

Supplementary Figure S3: The ribosomal protein abundance in the nuclear fraction is dependent on the mTOR kinase activity. MDA-MB-435 cells incubated with 500 nM of pp242 for the indicated time were lysed with the following fractionation to the cytoplasmic (Cyt) and nuclear (SENF) fractions. Both fractions were analyzed by immunoblotting with the indicated antibodies. From a set of the nuclear proteins only the levels of rpS3, rpS6 and rpL26 have been dramatically reduced in SENF following 1 or 3 hour treatment of cell with pp242. The levels of Nup98, FBxO22, topoisomerase II, EHD were not sensitive to the pp242 treatment.

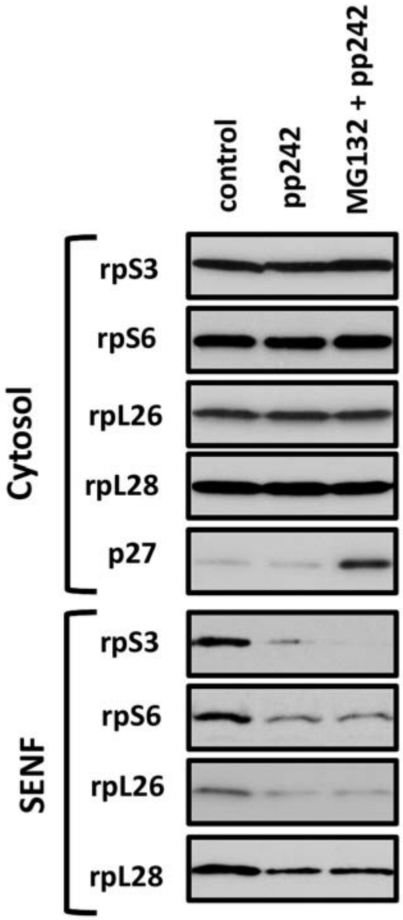

Supplementary Figure S4: A proteosomal inhibitor MG132 has no effect on the mTOR-dependent effect on the nuclear abundance of ribosomal proteins. MDA-MB-435 cells were treated with 10 mM of MG132 for 2 hr and incubated with or without pp242 (500 nM) for another hour. Following treatment, cells were lysed with the following sub-cellular fractionation. The cytosolic and nuclear fractions were analyzed by immunoblotting. p27 was examined as a marker for detection of the proteosomal inhibition by MG132.

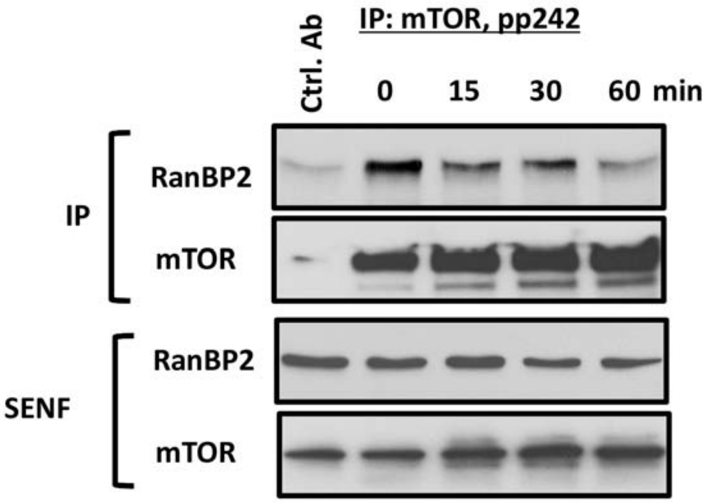

Supplementary Figure S5: The kinase activity of mTOR is required for association of mTOR with RanBP2 in the nuclear fraction. The actively growing MDA-MB-435 cells were treated by 500 nM pp242 for the indicated times. The intact nuclei were isolated and lysed in the buffer E to obtain the SENF fractions. The mTOR immunoprecipitates were prepared from the SENF fractions and analyzed by immunoblotting. Abundance of the indicated proteins in SENF is presented in a lower panel. Following pp242 treatment the mTOR translocation to SENF is observed similar to the observation described in the Figures 3A and 3B.

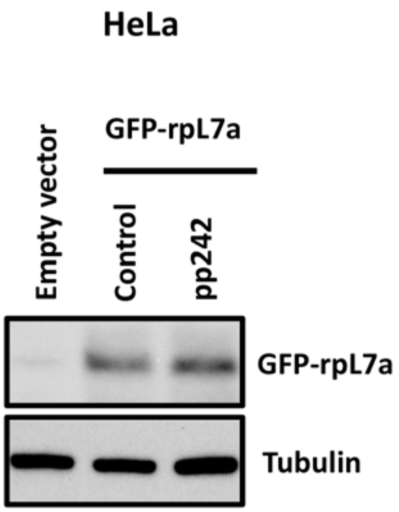

Supplementary Figure S6: Inhibition of the mTOR kinase activity by pp242 does not suppress expression of the GFP-rpL7a protein. HeLa cells were transfected with the GFP-rpL7a expression plasmid and incubated with or without 500 nM pp242 for 5 hrs. The recombinant protein expression was examined by immunoblotting for the levels of GFP-tagged protein and tubulin.

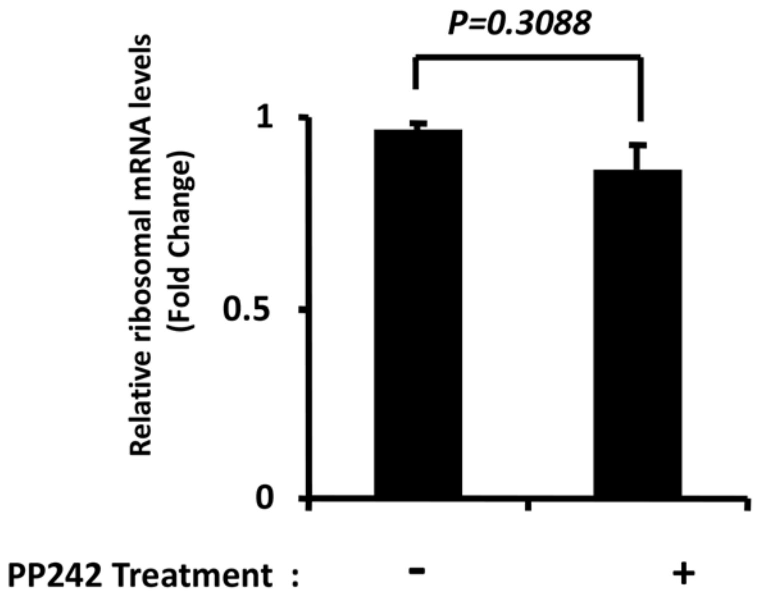

Supplementary Figure S7: A transient inhibition of the mTOR kinase activity has no substantial effect on expression of the ribosomal mRNA. MDA-MB-435 cells were incubated with or without 500 nM pp242 for 1 hr and cells were extracted to purify total RNA. Abundance of 18S ribosomal mRNA has been analyzed by qRT-PCR analysis. Normalization was done against GAPDH mRNA. Results represent the mean and standard deviation of three independent experiments.

Supplementary Table S1. Quantitative analysis of the nuclear co-localization of mTOR and RanBP2 (A) Whole cell area. **(B)** Three representative of the co-localized areas. Images obtained with confocal laser scanning microscope (LSM710, Zeiss, Jena, Germany) (x63) were analyzed using ZEN 2011 software that calculated Pearson's correlation coefficient. This is a standard technique in pattern recognition that matches one image to another in order to describe the degree of overlap between two patterns and calculates the overlap coefficient, according to Manders. The overlap coefficient indicates an overlay of the signals and, thus, represents the true degree of co-localization. The average overlap coefficient for the mTOR and RanBP2 co-localization is 0.8±0.03.

A

Mean Intensity Ch2-T3	Mean Intensity ChS1-T2	Standard Deviation Ch2-T3	Standard Deviation ChS1-T2	Colocalization Coefficient Ch2-T3	Colocalization Coefficient ChS1-T2	Weighted Colocalization Coefficient Ch2-T3	Weighted Colocalization Coefficient ChS1-T2	Overlap Coefficient
76.7	23.2	31.5	11.0					
23.2	65.7	13.3	24.4					
107.8	97.1	45.4	42.4	0.761	0.720	0.817	0.791	0.77

B

Relative Area (%)	Mean Intensity Ch2-T3	Mean Intensity ChS1-T2	Standard Deviation Ch2-T3	Standard Deviation ChS1-T2	Colocalization Coefficient Ch2-T3	Colocalization Coefficient ChS1-T2	Weighted Colocalization Coefficient Ch2-T3	Weighted Colocalization Coefficient ChS1-T2	Overlap Coefficient
5.9	146.4	28.4	40.8	8.6					
0.0	35.0	97.4	6.5	33.1					
69.9	161.1	111.8	41.6	44.6	0.922	0.999	0.929	0.999	0.83
8.9	123.8	27.5	36.8	9.1					
0.2	36.8	88.1	4.7	28.1					
67.9	136.5	104.5	40.0	43.7	0.884	0.998	0.894	0.998	0.8
12.7	94.8	25.3	33.3	10.0					
2.8	29.8	70.7	11.3	26.2					
53.2	116.1	98.9	41.2	43.3	0.807	0.949	0.836	0.963	0.79
